# Otolaryngology, Head and Neck Surgery

**DOI:** 10.1155/2014/625601

**Published:** 2014-12-28

**Authors:** Tsung-Lin Yang, Robert L. Ferris, Tetsuya Ogawa, Peter Hwang, Michael Tong, Mu-Kuan Chen

**Affiliations:** ^1^Department of Otolaryngology, National Taiwan University Hospital, Jen-Ai Road, Taipei 100, Taiwan; ^2^Department of Otolaryngology, Head and Neck Surgery, University of Pittsburgh Medical Center, Pittsburgh, PA 15213, USA; ^3^Department of Otorhinolaryngology, Head and Neck Surgery, Aichi Medical University School of Medicine, Aichi 480-1195, Japan; ^4^Department of Otolaryngology, Head and Neck Surgery, Stanford University School of Medicine, Stanford, CA 94305, USA; ^5^Department of Otolaryngology, Head and Neck Surgery, Chinese Hong Kong University, Hong Kong; ^6^Department of Otorhinolaryngology, Head and Neck Surgery, Changhua Christian Hospital, Chung Shan Medical University, Changhua, Taiwan

The medical fields of otolaryngology and head and neck are evolving rapidly. For the past years, much new knowledge and many techniques have been introduced into the basic researches and clinical practices of otolaryngology and head and neck for a comprehensive understanding of diseases and practical clinical applications. Just recall the recent development of many medical devices designed for enabling techniques and surgery, including the newly designed hearing aids and coagulative surgical instruments; the trend of newly developed technologies is likely to be a constant flow. In addition, many distinct diagnostic modalities and research approaches have also been invented to investigate the underlying phenomenon and mechanisms of diseases. The cornucopia of all novel technologies and approaches serve as important blessings for both doctors and patients in the fields of otolaryngology and head and neck. This special issue is to showcase the diversity and advances in recent progress that contributes to the different subspecialties of otolaryngology and head and neck surgery.

Recent advances both in basic and clinical studies have introduced new concepts and technologies to be applied in otolaryngology and head and neck surgery. In this special issue, several special topics regarding the applications of hearing devices and vestibular evoked myogenic potential, the surgical tools for potentiating the procedure of thyroidectomy, and identification of the novel therapeutic agents and underlying mechanism of head and neck cancer will be presented. One article of each field will be selected as the representative example of the progress. These articles will demonstrate the current advance of medical development in otolaryngology and head and neck surgery.

An article will discuss the role of screening of cognitive function in old adults because of the efficacy of using hearing aid assistance. Previous studies had found that hearing loss is associated with poorer cognitive function. This study examined cognitive function in elderly hearing aid users. The study investigated whether the screening cognitive function should be considered in the individuals with hearing impairment. On the other hand, for the adult patients with unilateral microtia and atresia, many devices of implantable hearing amplification have been created for hearing ability rehabilitation. An article in this special will give a comprehensive review of the development, the progress, and the technical advantages of the different types of implantable hearing devices available. After understanding the pros and cons of each specific type of devices, it is easy to make the clinical decision of recommending a device to match the need of patients.

Apart from the hearing ability, the vestibular function, another essential component of the 8th cranial nerve, has also drawn much attention of researchers and clinicians. An article included in this special issue will demonstrate the use of skull tap vestibular evoked myogenic potentials of diagnosing cerebellopontine angle tumor. The tap vestibular evoked myogenic potentials (tap VEMPs) are another method of inducing VEMPs by tapping method in contrary to the traditional one of using auditory stimulation. In the diagnosis and pretreatment evaluation of cerebellopontine angle tumor, the involvement and the extent of tumor are critical for the following treatments and can be clearly identified by clinical physiological examinations. In this article, the tap VEMPs could be successfully induced in some of the recruited patients. It shows a potential of clinical utility of combining both AC VEMP and tap VEMPs in the evaluation of patients suffering from cerebellopontine angle tumors.

With the innovation of many tools of basic research, the understanding of molecular and cellular mechanism has achieved a profound advancement in the head and neck cancer. An article in this special issue will address the importance of finding novel and potential therapeutic reagents that target the mitochondria-dependent pathway. Tanshinone IIA (Tan IIA) is an active phytochemical retrieved from the dried root of* Salvia miltiorrhiza *Bunge. It has been documented to be an antiproliferative reagent on various human cancer cells. This research aims to evaluate its role in nasopharyngeal carcinoma. By showing the effects of antiproliferation and apoptosis induction, Tan IIA is believed to be a potential candidate of exploring the anticancer drugs. On the other hand, bleeding is always a major concern in the procedure of thyroidectomy. Many distinct skills have been developed to control bleeding, and multiple devices and surgical instruments have been created to facilitate the hemostasis. Ligasure and bipolar thermofusion are two surgical tools that have been widely used to achieve hemostasis during thyroidectomy. In an article included in this special issue, the comparison between these two techniques will be addressed when more than 2122 lobectomies were performed within 8 years. The experience earned from the large patient population provides convincing evidence of using these distinct surgical tools for bleeding control of thyroidectomy.

The researches and development of new technologies and knowledge of the field of otolaryngology and head and neck surgery are committed to improving excellent medical care in all subspecialties to the patient groups worldwide. It is also committed to educating the health provider and investigators in the basics and the clinical practices of otolaryngology and head and neck surgery for the further progress in each subspecialty. The current knowledge and understanding have led to the exploration of new mechanism underlying the diseases and also development of novel therapies, surgical techniques, and tools to change the way of clinical practices. It is believed that the evolvement of medical researches and clinical health care may substantially improve the clinical outcomes and life quality of patients.

*Tsung-Lin Yang* *Robert L. Ferris* *Tetsuya Ogawa* *Peter Hwang* *Michael Tong* *Mu-Kuan Chen*

